# Assessment of community knowledge, practice, and determinants of malaria case households in the rural area of Raya Azebo district, Northern Ethiopia, 2017

**DOI:** 10.1371/journal.pone.0222427

**Published:** 2019-10-15

**Authors:** Kebede Tesfay, Mekonnen Yohannes, Fitsum Mardu, Brhane Berhe, Hadush Negash

**Affiliations:** 1 Unit of Medical Parasitology and Entomology, Department of Medical Laboratory Sciences, College of Medicine and Health Sciences, Adigrat University, Adigrat, Ethiopia; 2 Medical Parasitology and Entomology Unit, Institute of Bio-medical Science, College of Health Science, Mekelle University, Mekelle, Ethiopia; 3 Unit of Microbiology Department of Medical Laboratory Sciences, College of Medicine and Health Sciences, Adigrat University, Adigrat, Ethiopia; Instituto Rene Rachou, BRAZIL

## Abstract

**Background:**

In Ethiopia malaria is one of the leading causes of outpatient visits and admission. Still, it remains a major cause of morbidity and mortality in the study area. Therefore, this study was aimed to assess the knowledge, practice, and determinant of malaria case households in rural areas of Raya Azebo district, Northern Ethiopia.

**Method:**

A community-based cross-sectional survey was conducted in the selected villages of Raya Azebo district from January to June 2017. A multi-stage random sampling method was employed to select a total of 422 study households. Data was collected using a semi-structured questionnaire. The household head was interviewed face to face. Logistic regression analysis was used to determine the determinant of malaria cases households.

**Result:**

A total of 412 (97.6) of the respondents had ever heard about malaria. About 63% of households recognized the causes of malaria to be a mosquito bite. Around 173 (41%) of the study households had been treated for malaria within a year of data collection. This study also revealed that the presence of mosquito breeding sites near to home, bed bug infestation, outdoor sleep due to bed bugs and household with poor bed net practicing were significantly associated with malaria case households.

**Conclusion:**

Although the overall knowledge on malaria transmission, symptoms, and the preventive measure was relatively good, the rate of household insecticide-treated net coverage and utilization were reported low in the area. Therefore, the distribution of adequate bed net with community-based education is a key intervention to promote household insecticide-treated net utilization. In addition, an effective bed bug management strategy is necessary to overcome the outdoor sleeping habit of the community in the area.

## 1. Introduction

Malaria remains a major public health problem in Ethiopia. In 2009/2010, malaria was the leading cause of outpatient visits and health facility admissions, accounting for 14% of outpatient visits and 9% of admissions in the country **[1].** The Federal Ministry of Health reported 4,068,764 malaria cases to the World Health Organization as recorded in the 2011 World Malaria Report **[2]**. Malaria transmission exhibits a seasonal and unstable pattern in Ethiopia, with transmission varying with altitude and rainfall. The major malaria transmission season in the country is from September to December, following the main rainy season. In general, 75% of the landmass and around 68% population of Ethiopia considered at risk of malaria, which corresponds to areas below 2,000m altitude. *Plasmodium falciparum* and *Plasmodium vivax* are the most dominant malaria parasites in Ethiopia **[3, 4].**

The scale-up of malaria prevention and control intervention was increased in the last decade in Ethiopia. According to the Ethiopian malaria indicator survey of 2015, 71% of households are protected either by owning an insecticide-treated net or having received indoor residual spray (IRS). As a result, the prevalence of malaria parasite was reduced from 1.3% in 2011 to 0.5% in 2015 **[5, 6]**. On the other hand, the ongoing malaria control interventions are faced with many challenges including under-utilization of intervention, not provide sufficient and quality data, gaps in service delivery and health system weaknesses **[3]**. In addition, poor community awareness toward malaria and insecticide-treated nets (ITN) practicing were reported as a main problem in the country **[7].**

Malaria is a major cause of morbidity and mortality in Tigrai. The dynamics of malaria transmission in Tigrai is similar to that of the country including in intensity, distribution, seasonality, type, and proportion of species involved, and types of malaria control interventions **[8, 9]**. As in the rest of the country, the scale-up of malaria control activities in the past decade has resulted in a significant reduction in malaria prevalence in Tigrai. According to health management information system (HMIS) data of Tigrai, the proportion of total out-patient department (OPD) visits, admissions, and deaths due to malaria decreased from 20.5%, 10.5% and 5.1% in 2011/2012 to 11.6%, 4.4% and 1.9% in 2014/15 respectively in the region. However, despite some reductions in prevalence, malaria remains one of the leading causes of morbidity in the region ranked second in 2014/15 with 296,785 (11.55%) cases as an outpatient and 5,417 inpatient cases **[10]**.

A number of factors influence the epidemiology of malaria in Tigrai. Environmental factors mainly associated with the ongoing efforts to alleviate the impact of climate change, such as rainwater harvesting, irrigation, and resettlement programs had their own negative impact on malaria transmission in the region **[11].** A case in point is the outcome due to the construction of thousands of ponds and dams in the Raya Valley in the past decade has resulted in an increased risk of malaria transmission with incidence and prevalence rates recorded high in villages with ponds in the Raya Valley **[8, 11]**. Similarly, the construction of soil conservation and rainwater harvesting structures for water resources conservation have also resulted in malaria outbreaks in some parts of the region **[12]**. These together with the emerging threat of insecticide resistance, outdoor and early vector biting are expected to bring changes in infectious disease dynamics, creating a high level of risk to the communities living in the area **[13]**.

Therefore, this study was conducted to assess the community knowledge, practice, and determinants of malaria case households in the rural area of Raya Azebo district, Northern Ethiopia.

## 2 Matreials and methods

### 2.1 Study area and population

**Fig 1:**
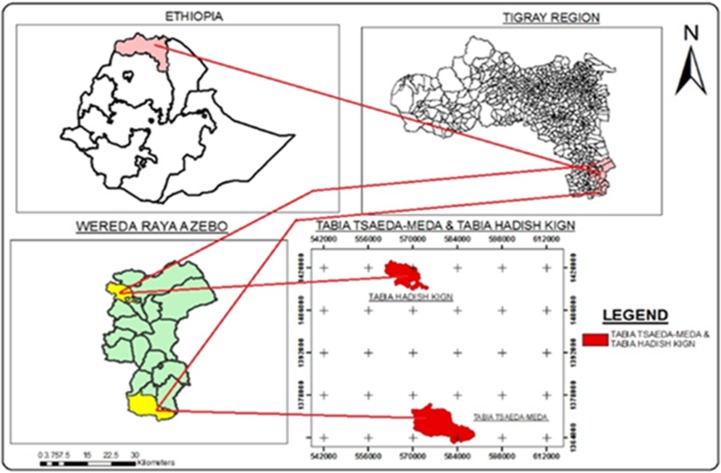
Map of the study area Raya Azebo district.

A community-based cross-sectional study was conducted in the selected rural areas of Raya Azebo district, Northern Ethiopia from October 2016 to June 2017. Raya azebo is one of the district of Tigrai regional state which is located 652 km from the capital city of Addis Ababa. A total of 135, 870 peoples are living in the district, of which 67,687 are male and 68,173 female. More than 85% of the population are rural residents(Fig 1).

The administrative of the district is sub-divided into 20 Kebeles (or sub-districts). It is lying at an altitude range of 930 to 2,300 meters above sea level. Each Kebeles are divided into 3.8 clusters of villages locally known as kushets. Regarding to health facility, one primary hospital and eight health centers are found in the district. Each village found under the district has one health post **[14].**

The total area of the district is 176,210 hectares. From this, most of the area is cultivable land. A thousand of water harvesting ponds and dams were constructed in the last decade in the area. The district is characterized by having a bimodal type of rainfall pattern with light rains during the February to April and heavy rains between July to September. The mean annual rainfall is about 300–724 mm with mean daily maximum and minimum temperatures of 23.44°C and 19.64°C, respectively in the valley. Ninety percent of the district is described as “midland” (1500-2300m) and 10% is considered to be “lowland”(< 1500m) **[15].**

### 2.2 Sample size and Sampling procedure

The sample size was calculated using a single population proportion formula (N = Z^2^α/2 × P (1−P)/ m^2^) at 95% confidence level, 5% margin of error, and 50% population proportion. Since there is no similar study carried out in the area, we assumed 50% of the population as having knowledge of malaria transmission and prevention methods. We also considered a 10% non-response rate. Therefore, a total of 422 households (HH) head was included in the study.

A multi-stage random sampling method was used to enroll in the study participants. The district has eight health center catchment areas. One health center is available to serve a cluster of Kebeles called catchment area. Based on the assessment malaria profile of the district, the two most malaria-affected Kebeles (Tsaeda- meda, and Hadish-kign) were selected. The total households for each village were available in their nearest health center or health post which is stored as a family card folder. Based on this, there were 1586 and 1505 households in Tsaeda-meda and Hadish-kigni Kebeles, respectively. Finally, the total sample size was proportionally allocated to each Kebele.

### 2.3 Data collection

A standard semi-structured questionnaire was used to collect data about the scio-demographic characteristics, knowledge, and practice of respondents related to malaria transmission, symptoms, and prevention methods. In addition, we have collected information on ITN coverage, utilization, history of malaria and treatment-seeking behavior. The questionnaire was first designed in English and translated to Tigrigna (Native language of participants) and pre-tested on non- selected Kebeles for assessing its validity, correctness, and question broadly. After that, the questionnaire was modified if necessary. Three nurses have participated during data collection. The training was given to the data collector. The household head either the father or mother was openly interviewed face to face. In case the father or mother is absent, another adult member of the household was interviewed. All household information was collected without prompt the participants. The principal investigator was supervising daily the work of data collectors.

### 2.4 Measurement of Knowledge and practice

A series of questions were used to measure the knowledge and practice level of households on malaria transmission and preventive measures. A total of four questions were designed related to the cause, transmission, signs and symptoms, preventive measure of malaria. Questions were marked 1and 0 for right and wrong answers respectively. If respondents properly answered for those questions like the cause of malaria is to mosquito bite, the way of malaria transmission (through biting of infected mosquito), properly mentioned more than three sign and symptom of malaria (Chills, fever, headache) and properly mentioned the main malaria prevention method (using of bed net, house spraying with chemical), we considered right answer. But if a respondent fails to answer properly those questions we have considered wrong response. The overall knowledge of the study participant was measured by calculating the total score for each question. The participant who scored more than the mean average (>50%) of the total questions were considered as knowledgeable (good knowledge). In addition, two questions; 1. Bed net used every night, 2. Irregular use or not use at all was designed to measure participant ITNs utilization. The participant who used bed net regularly or every night were considered good ITNs practitioners. But these irregularly or not use bed net at all were considered as poor ITN practitioners.

Based on this measurement of knowledge and practice of participant, two independent variables (malaria knowledge status and ITN utilization) were designed for the analysis.

### 2.5 Data analysis

Data were analyzed using SPSS version 21. Descriptive statistics were computed for all variables. The Knowledge of respondent to malaria symptom, transmission, preventing methods are presented as frequency and percentages using tables. Binary logistic regression analysis (bivariate) was carried out to determine the association between household having malaria case (dependent variable) with socio-demographic factors, malaria knowledge, prevention method, and environmental variables. Variables with a p-value less than 0.05 were further entered into a multivariate logistic regression to test the independent association. Finally, Odds ratio, CI = 95%, and P-value = <0.05 were computed to measure the strength of the association.

## 3. Results

### 3.1 Socio-demographic characteristics

In this study, a total of 422 household heads have participated with a 100% response rate. The mean age of the respondents was 39.04 years (SD+/-11.8) and ranged from 18–70 years. Out of the total respondents, 279 (66%) were females. Nearly half (51%) of the participants were in the age category of >36 years, followed by 29.6% belong to 26–35 years. All of the respondents (100%) were rural dwellers (Table 1).

**Table 1:** Socio-demographic characteristics of respondents showing the number of malaria case household and bivariate regression analyses in Raya Azebo district, Tigrai, Northern Ethiopia, 2017. (n = 422).

Socio-demographic variable		Frequency(%), n = 422	Malaria case HH		Crude OR(95% CI)	P–value
			Yes	No		
Sex	Male	143(34)	68	75	1.441(0.804–2.581)	0.220
	Female	279(66)	105	174	1.00	
Age categories	18–25	82(19.4)	23	59	0.547(0.247–1.212)	0.137
	26–35	125(29.6)	74	51	1.490(0.790–2.812)	0.218
	>36	215(51)	88	127	1.00	
Residence	Rural	422(100)	173	249	NA	
Occupation	Farmer	139(33)	65	74	1.244(0.359–4.311)	0.730
	House wife	258(61.1)	98	160	0.862(0259–2.868)	0.808
	Merchant	25(5.9)	10	15	1.00	
Educational status	Illiterate	303(71.8)	119	184	0.483(0.104–2.239)	0.375
	Can write and read	21(4.9)	15	6	1.750(0.233–13.159)	0.587
	1- 8^th^ finished	84(19.9)	33	51	0.480(0.095–2.433)	0.375
	High school and above finished	14(3.4)	8	6	1.00	
Religion	Orthodox Christian	344(81.5)	141	203	0.958(0.469–1.956)	0.907
	Muslim	78(18.5)	33	45	1.00	
Family size /HH	1–4	170(40.3)	64	106	0.894(0.290–2.753)	0.846
	5–8	221(52.4)	98	123	1.200(0.399–3.607)	0.745
	>9	31(7.3)	12	19	1.00	
Walking distance home to health facility in minute	<60 minute	180(42.7)	84	96	1.467(0.837–2.571)	0.180
	>60 minute	242(57.3)	90	152	1.00	
Type of house structure	Tukul with thatched roof	213(50.5)	86	127	0.961(0.552–1.673)	0.887
	Corrugated with iron	209(49.5)	88	121	1.00	
Income of household per month in birr	Low(<660)	190(45)	84	106	0.574 (0.121–2.173)	0.83
	Medium (1320–2980)	225(53.3)	90	142	0.470(0.100–2.206)	0.339
	High (>3300)	7(1.7)	0	0	1.00	
Sources of information	Yes	230(54.5)	94	136	0.983(0.563–1.715)	0.952
	No	192(45.5)	80	112	1.00	
Presence of house bed bug	Yes	266(63)	129	137	2.308(1.262–4.220)	0.007*
	No	156(37)	45	111	1.00	
Sleeping out-door due to bed bug	Yes	186(44)	49	137	2.758(1.444–5.265)	0.002*
	No	236(56)	45	191	1.00	
Mosquito breeding site near home stead	Yes	266(63)	143	123	1.976(1.001–3.903)	0.045*
	No	156(37)	31	125	1.00	

Key = *statistically significance, P <0.05. Abbreviation, COR = Crude Adds Ratio, HH = household, CI = confidence interval, NA = No analysed

### 3.2 Knowledge and practices regarding malaria

The majority (97.6%) of the respondents have ever heard about malaria. About 63% of the respondents mentioned biting mosquito as the main way of malaria transmission. Similarly, 63% of the participants mentioned chill as the main malaria symptom, followed by fever (51.2%). Nearly 88% of the respondents believed that malaria is a preventable disease. Of these, 225 (53.3%) thought that the use of a mosquito bed net prevents malaria transmission while 148 (35%) replied environmental management as a malaria prevention method (Table 2).

**Table 2:** Household knowledge and practice regarding malaria showing bivariate analysis in selected rural area of Raya Azebo district, Northern Ethiopia, 2017.

Variables		Frequency(%)	Malaria case household		Crude OR(95% CI)	P-value
			Yes	No		
**Ever heard about malaria**	Yes	412(97.6)	173	239	1.243(0.115–0.318)	0.735
	No	10(2.4)	4	6	1.00	
Mode of malaria transmission		65(15.4)	24	41	2.361(0.844–6.605)	0.102
Environmental change		31(7.4)	12	19	0.354(0.082–1.533)	0.165
Don't know		60(14.2)	21	39		
Mosquito bite		266(63)	117	149	0.364(0.092–1.644)	0.175
**Malaria symptom**						
Chills(n = 422)	Yes	266(63)	102	164	0.732(0.431–1.298)	0.286
	No	156(37)	70	86	1.00	
Fever(n = 422)	Yes	216(51.2)	102	114	0.603(0.344–1.056)	0.77
	No	206(48.8)	72	134	1.00	
Headache(n = 422)	Yes	207(49)	100	107	2.130(1.211–3.748)	0.009*
	No	215(51)	70	145	1.00	
Joint pain(422)	Yes	162(38.4)	74	88	1.333(0.754–2.354)	0.332
	No	260(61.6)	79	181	1.00	
Loss pf appetite(422)	Yes	84(19.9)	33	51	0.890(0.442–1.792)	0.745
	No	338(80.1)	124	214	1.00	
Sweating(422)	Yes	50(11.8)	21	29	1.057(0.595–1.870)	0.851
	No	372(88.2)	152	220	1.00	
**Does malaria cause death**	Yes	383(90.8)	166	217	2.866(0.916–8.962)	0.07*
	No	39(9.2)	8	31	1.00	
**Malaria can be cured**	Yes	400(94.8)	168	232	1.935(0.498–7.517)	0.34
	No	22(5.2)	6	16	1.00	
**Malaria can be prevented**	Yes	371(87.9)	150	221	0.789(0.225–2.442)	0.68
	No	51(12.1)	26	25	1.00	
**Malaria prevention method(n = 422)**						
Use of LLINs	Yes	225(53.3)	101	124	1.339(0.766–2.341)	0.306
	No	197(46.7)	74	123	1.00	
Apply environmental sanitation	Yes	148(35)	53	95	1.657(0.833–3.2960	0.15
	No	274(65)	121	153	1.00	
Keeping personal hygiene	Yes	115(27.2)	41	74	0.728(0.385–1.371)	0.324
	No	307(72.8)	133	174	1.00	
House spraying with chemical	Yes	84(19.9)	43	41	0.718(0.398–1.296)	0.324
	No	338(80.1)	131	207	Jan-00	
**Malaria knowledge score**	Good	300(71)	122	178	1.066(0.578–1.967)	0.837
	poor	122(29)	70	52	1.00	
**Houshold own LLINs**	Yes	201(47.6)	76	125	0.769(0.441–0.856)	0.05*
	No	221(52.4)	98	123	1.00	
**Source of ITNs(n = 201)**						
Government		201(100)	77	124	NA	
**Frequency of bed net usage(n = 201)**						
Regular use(Bed net use evry night)		84(41.8	12	72	1.219(0.441–1.321)	0.356
Irregular use(Missed some times)		78(38.8)	29	49	0.829(0.380–1.801_	0.638
Not using		39(19.4)	16	23	1.00	
**Family member most often used bed net(n = 201)**						
Women	Yes	74(37)	28	46	0.777(0.395–1.528)	0.465
	No	126(63)	50	77	1.00	
Under five children	Yes	59(29.4)	22	37	0.760(0.450–1.575)	0.461
	No	142(70.6)	56	86	1.00	
Father	Yes	55(27.2)	20	35	1.175(0.818–1.690)	0.383
	No	146(72.8)	59	87	1.00	
Pregnant women	Yes	7(3.6)	2	5	2.565(0.226–1.364)	0.381
	No	194(96.4)	73	121	1.00	
All member	Yes	6(2.8)	2	4	0.420(0.042–4.174)	0.432
	No	195(97.2)	77	178	1.00	
**Any one slept in bed net last night(n = 201)**	Yes	121(60.2)	17	104	0.857(0.450–1.631)	0.482
	No	80(39.8)	42	38	1.00	
ITNs practicing score	Good	177(42)	25	152	0.344(0.144–0.826)	0.017*
	poor	245(58)	182	63	1.00	
IRS using in last 12 month	Yes	354(85)	146	208	0.968(0.446–2.099)	0.934
	No	68(15)	29	39	1.00	

Key = *statistically significance, P <0.05. Abbreviation, COR = Crude Adds Ratio, H = household, CI = confidence interval, NA = No analyzed

Over half (52.4%) of the households claimed that they had no ITNs. The mean number of ITNs owned per household was 1.35 (+/-0.35) which all they got from government health facilities. The majority (60.2%) of the ITN owners claimed that they slept under a bed net the night before the interview. Most of the household ITN owners (37%) stated that women were given priority to sleep under bed net followed by under-five children (29.4%). Nearly (41.8%) of the respondents stated that they utilize bed net regularly /every night. Around 38.8% of respondents stated they were missed using bed net regularly. The rest 19.4% of the HH claimed they do not use bed nets. According to the household respondents, indoor residual house spraying is one of the most important malaria prevention methods in the study area. Out of the total HHs, 85% stated that their house was sprayed with chemicals in the last 12 month (Table 2)

### 3.3 History of malaria and treatment-seeking behavior

Of the total respondents, 173(41%) stated that at least one member of the household was treated for malaria in the last 12 months. A maximum of four malaria cases was reported per household. Nearly 57 (32.9%) of the respondents claimed that young adolescents were the most affected group by malaria followed by the under-five children 52 (30%). Most of (96%) malaria casehouseholds were treated in governmental health facilities.

### 3.4 Determinants of malaria case household

In this study, we have assessed the determinant factors associated with households having a history of malaria cases. Presence of mosquito breeding site near to home and large scale of bed bug infestation was stated as determinant factors with malaria case households. A total of 63% household was mentioned they had mosquito breeding site near their home such as; ongoing construction of a new railway, many ponds, and huge agricultural area with irrigation. Similarly, 63% of the households had a bed bug infestation in their home. Due to the bed bug infestation, 70% of the households had been sleeping outdoor at night (Table 1).

Logistic regression analysis (bivariate and multivariate) was used to determine the association between socio-demographic variable, malaria prevention methods, and environmental factors with malaria case households.

Environmental and socio-demographic factors like presence of mosquito breeding site near home, HH possess of long-lasting insecticidal treated nets, bed bugs infestation of house, sleeping outdoors due to bed bugs, and poor ITN utilization was significantly associated with malaria case household. In addition, these respondents mentioned properly headache as malaria symptom was significantly associated with malaria case household (Table 1, Table 2)

All variables with a p-value less than 0.05 in bivariate analysis were further analyzed by multivariate logistic regression. As a result, presence of mosquito breeding site near to home, bed bugs infestation in the house, outdoor sleeping and poor practicing of ITN and respondents mentioned properly headache as malaria symptom was significantly associated with malaria case household. Households who had mosquito breeding sites near their home were 2 times more likely to have a history of malaria than that living far-away from mosquito breeding sites [AOR = 2.001, CI = 1.123–9.339]. Similarly, households with bed bug infestation were 2.098 times more likely to have malaria cases than those HHs without bed bugs [AOR = 2.098, CI = 1.131–3.892]. Household with good ITN practice were 0.344 (34.4%) times less chance to be exposed to malaria than those poor ITN practitioner [AOR = 0.336, CI = 0.135–0.832] (Table 3).

**Table 3:** Multivariate analyses of determinant factors among malaria case household in the rural area of Raya Azebo district, Northern Ethiopia,2017 (n = 422).

Variable		Frequency(%)	Malaria case households		Crude OR(95% CI)	P–value	AOR(95% CI)	P–value
			Yes	No				
Mentioned Headache symptom	Yes	207(49)	100	107	2.130(1.211–3748)	0.009	2.046(1.128–3.713)	0.024*
	No	215(51)	70	145	1.00		1.00	
Household own LLINs	Yes	201(47.6)	76	125	0.769(0.441–0.856)	0.05	0.987(0.566–1.245)	0.894
	No	221(52.4)	98	123	1.00		1.00	
Presence of house bed bug	Yes	266(63)	129	137	2.308(1.262–4.220)	0.007	2.098(1.131–3.892)	0.019*
	No	156(37)	45	111	1.00		1.00	
Sleeping out-door due to bed bug	Yes	186(44)	49	137	2.758(1.444–5.265)	0.002	2.493(1.282–4.848)	0.007*
	No	236(56)	45	191	1.00		1.00	
Mosquito breeding site nearhomestead	Yes	266(63)	143	123	1.976(1.001–3.903)	0.045	2.001(1.123–9.339)	0.05*
	No	156(37)	31	125	1.00		1.00	
ITN practicing score	Good	177(42)	25	152	0.344(0.144–0.826)	0.017	0.336(0.135–0.832)	0.018*
	Poor	245(58)	182	63	1.00		1.00	

Key = *statistically significance, P <0.05. Abbreviation, COR = Crude Adds Ratio, AOR = Adjusted Odds Ratio, HH = household, CI = confidence interval

## 4. Discussion

This study was aimed to assess the household awareness and practice towards malaria transmission and preventive measures in Raya Azebo district. In addition, we also have assessed the determinants of malaria case households in the selective villages of the study area.

The finding showed, that the general awareness of malaria was high among the household in the rural area of Raya Azebo district, almost (97.6%) had heard about malaria. This is expected because malaria is considered the major cause of morbidity and mortality in the study area **[16].** This awareness was lined with different studies conducted in Swaziland **[17]** and Nigeria **[18].** About 63% of the respondent mentioned mosquito bite was the main way of malaria transmission, which was lower than from studies conducted in Ethiopia **[19, 20]** and Iran **[21].** The difference may be due to the variation of respondent characteristics like the educational level. Most of the respondents (71.8%) in our study were illiterate, unlike those studies in Ethiopia and Iran (56.1%, 5.6%, 37.3%) respectively **[19, 20, 21]**. Sources of information may be the other reason for malaria transmission awareness difference, which about 45.5% of the households have not source of information for malaria in our study.

The present study revealed, most of the participants were able to identify the main sign and symptoms of malaria. Chill, fever, headache were among the most mentioned malaria symptom in this study. Similar results were found from different KAP studies including Iran, Colombia, Bangladesh, and Tanzania **[21–24]** respectively. The finding also supported by other studies conducted in Ethiopia **[25–27]**. In addition, this good awareness in our study was supported by the Center of Disease Control and Prevention (CDC) listed malaria sign and symptom **[28].**

Regarding malaria prevention methods, 87.9% of the respondents perceived that malaria is a preventable disease. This is consistent with the study conducted in Abeshge, Ethiopia **[29]**. However, this awareness is lower than a study reported in Pawe, Ethiopia **[30]** the and South Africa **[31]**. Use of mosquito bed net, house spraying, and environmental management was among the frequently mentioned malaria prevention measures by the study participants. This finding is consistent with other studies in conducted Tanzania [24] and Iran **[32].**

According to our result, the distribution of ITNs was low in the study area. More than half (52.4%) of the study HH have not to bed net during our observation. In fact, Ethiopia has made a significant step in expanding the coverage of ITNs throughout the country. From 2014 to 2016, the Federal Ministry of Health (FMOH) distributed 29.6 million long-lasting insecticidal nets (ITNs) to protect all peoples living in the area with ongoing malaria transmission **[33].** The country has also targeted to eliminate malaria by 2020 by addressing 100% ITNs coverage in endemic areas [34]. Therefore, the ITNs coverage in this study can be considered low. In addition, Ethiopian malaria indicator survey (2015) showed that 64% of households in Ethiopia owned at least one ITN, which is higher than the present study (47.6%) **[35].** In some of the assessed households, we observed ITNs being used for other purposes such as storing agricultural hay, wood collection and tying of domestic animals. This improper use of nets might significantly reduce the coverage of ITNs in households.

In this study, the response of participants on ‘who slept under ITN last night’ and observation of ITN hanging on beds were used to measure HH bed net utilization. The majority (60.2%) of the ITN owners claimed that they slept under a bed net the night before the interview which was comparable with the Ethiopian malaria indicator survey reported 2015 (61%) **[35].** According to the respondents, women and under-five children are the most ITN users among the family members. Most of the household respondents stated women (37%) were the most ITN users in the area. Unexpectedly, pregnant women and under-five children, who are the most vulnerable group, were reported to be the lowest ITNs users (3.6%), (29.4%) respectively **[36].** This is lower than the finding reported from Arbaminch, Ethiopia **[37]** which was [35%, 40.3%] respectively. This low practicing of ITN could be due to, most of our study participants are illiterate, rural residence, and have not enough access to sources of information compared to other studies. Therefore, these respondent characteristics might have affected the awareness of ITNs practicing in the area. In addition, our study was conducted in the dry season in the area, which could be another reason for the low ITN utilization.

This study indicated that 41.8% of ITNs owners households were utilized bed net regularly or daily. In contrary, 19.4% of the ITNs owners were not using it properly during surveying. We have asked the respondents why they do not use ITN properly. They have raised different responses like no malaria season and not convenient to use ITN due to the hot condition of the house. In addition, we have observed most of the household ITN owners were using it for other purposes like for bedding, doormat, and women wrap of their hair. This showed us, it still needs strong effort to creating awareness on ITNs utilization in the area. Like ITNs utilization, indoor residual spraying was key malaria preventing method in Ethiopia. Houses of almost 85% of the household were sprayed within 12 months of the interview. However, this finding was inconsistent with Ethiopian national malaria program monitoring and evaluation planed 100% spraying of households from 2014–2020 **[38].**

We also have assessed the history of malaria and treatment-seeking behavior of each household. Of the total 422 respondents, 41% had at least one family member treated for malaria in the last 12 months before data collection. This implies malaria is still a big health problem in the area. Most of the malaria-affected groups were young adolescents (32.9%). The majority (96%) of households with malaria cases were treated in governmental health facilities. However, nearly 41.2% of the HH respondents with a history of malaria revealed that they sought treatment within one week after the onset of signs and symptoms. Only 38.8% of the respondents reported that they seek treatment within 24hrs. Traveling long distances to get health services could be the reason for this poor treatment-seeking behavior. Most of the respondents (57.3%) in our study spent >60 minutes to visits the health facility. The finding is supported by the study conducted in Thailand **[39].** Moreover, there was not an appropriate transportation service in the area. In addition, financial constraint and work overload or a caretaker could be a possible cause. This is comparable to a study conducted in Ghana **[40].**

This study found that the presence of large scale bed bug infestation was strongly associated with malaria case households [AOR = 2.098, CI = 1.131–3.892]. Whenever there is bed bug infestation, people prefer to sleep outdoor. Hence, they will easily become exposed to a mosquito bite. There is no previous published finding regarding the association between malaria and bed bug infestation. However, in countries like Ethiopia, bed bug infestation is a major public health concern. Bed bug causes significant psychological, social and economic impacts **[41].**

In addition, Presence of mosquito breeding site near home was significantly associated with malaria [AOR = 2.001, CI = 1.123–9.339]. This is maybe due to increased agricultural activities, construction of soil conservation, ongoing railway construction and rainwater harvesting structures (pond and dam) in the area which could play a great role in changing the pattern of malaria transmission. The finding showed that good utilization of ITNs has a preventive association with malaria case households [0.344(0.144–0.826)] which was comparable with study in Ethiopia **[42].** Accordingly, a household with good ITN practitioner was 0.344 (34.4%) times less chance to be exposed to malaria than those poor ITN practitioners.

## Limitation of the study

This cross-sectional study design can create some bias in this study. It only provides information about a certain point of times and the response of participants may not the same in a different season.

## Conclusion

The finding of this study revealed, that the overall knowledge on malaria transmission, symptom, and the preventive measure was relatively good in Raya Azebo district. However, the rate of household ITNs coverage and utilization was low in the area. Therefore, distributing adequate bed nets is advised to reduce the burden of malaria in the area. Moreover, strengthened community-based health education is a key intervention to promote household ITN utilization. This study provides evidence of some important determinants such as; increasing mosquito breeding sites near to home and large scale bed bug infestations were significantly associated with households having malaria cases. These results, to a large number of malaria cases household, were reported in the area during the survey. Hence, an effective bed bug management strategy is necessary to overcome the outdoor sleeping habit of the community in the area. Besides, community education on the impacts of a bedbug infestation should be strengthened in the area.

### Ethics statement

Ethical clearance was obtained from the Ethical Review Committee of Mekelle University, College of Health Sciences. A written support letter was also obtained from Tigrai Regional Health Bureau. In addition, each participant gave informed written consent. We kept the confidentiality of participants’ information.

## Supporting information

S1 Questionaires Questionaries for household survey of malaria knowledge and practice. (DOCX)
