# Supplementary material for: Assessment of community knowledge, practice, and determinants of malaria case households in the rural area of Raya Azebo district, Northern Ethiopia, 2017
Source: PLoS One. 2019 Oct 15;14(10):e0222427. doi: 10.1371/journal.pone.0222427 (PMC6794070; doi:10.1371/journal.pone.0222427)
Supplement: S1 Questionaires — (DOCX) [file pone.0222427.s001.docx]

## Questionnaire in English

Area of research conducted: ________________________________ HH selection number_____ ________________

Date: ________________

Address of HH

Kishet____________________

Kebelle ___________________

District __________________

**Part-I:** Questionnaire about socio demographic characteristics of Household in Raya Azebo district

NB; circle answer of respondents from the list

1. Sex: A. male B. female
2. Age __________________
3. Residence: 1. rural 2. urban
4. occupation:
5. farmer
6. merchant
7. student
8. government employee
9. unemployed
10. housewife
11. daily laborer
12. Religion of household head ;
13. orthodox
14. Islam
15. Catholic
16. Protestant
17. other
18. Household member families size:
19. 1-4
20. 5-8
21. >8
22. Distance from home to nearest health facilities in minute
23. <60 min
24. >60m
25. Do you or any member of your families living in the same compound own:
26. Television?
27. Radio?
28. Telephone
29. Books or magazine?
30. Type of house: NB; circle answer of respondents ○
31. tukul with thatched roof
32. rectangular corrugated with iron
33. Monthly income of HH
34. >150 US$(high)
35. 60-90 US$(medium)
36. >30US$(lower)

**Part-II:** household survey malaria risk factor evaluation

1. Have you ever heard about malaria? If your answer is ‘No’ Skip to Q ‘21’
2. Yes
3. No
4. Either malaria in this area?
5. Yes
6. No
7. Don’t No
8. How do you think malaria is transmitted? **NB ; circle that all apply, do not prompt**
9. Contacted with infected persons blood
10. Inhaling breath from infected person
11. Environmental change (cold to hot or hot to cold)
12. Flies after contacted with infected persons
13. Mosquito bite after biting an infected persons
14. From being in the rain
15. Eating maize cane
16. Other___________________________(specify)
17. Don’t know
18. How can you tell if a person have malaria? **NB: circle that all apply, without prompt**
19. Fever
20. Sweating
21. Headache
22. Chills/shivering
23. Poor appetite
24. Vomiting
25. Cough/breathing difficult
26. Diarrhea
27. Joint pain
28. Convulsion
29. Coma
30. Other _______________(specify)
31. Don’t know
32. Do you believe malaria can be prevented? If your answer is ‘No or Don’t know’ skip to Q ‘19’
33. Yes
34. No
35. Don’t know
36. How can you prevent yourself from getting malaria? **NB; circle all that apply do not prompt**
37. Isolating infected person
38. Avoiding movement or staying at home
39. Smoking the house with special smoking material
40. Spraying the house with chemicals
41. Covering body with mosquito nets at night
42. Taking chemoprophylaxis
43. Avoiding pond and dams
44. Cleaning bushy and swamp areas
45. Other________________________(specify)
46. Have you ever used one of the activities you just mentioned for malaria prevention?
47. Yes
48. No
49. Which activities have you ever used to prevent yourself from getting malaria? **Circle all that apply, do not prompt**
50. Isolating infected persons
51. Avoiding movement or staying at home
52. Smoking the house with special smoking material
53. Spraying the house with chemicals
54. Covering body with mosquito nets at night
55. Taking chemoprophylaxis
56. Avoiding pond and dams
57. Cleaning bushy and swamp areas
58. Do you believe that malaria can be cured through treatment?
59. Yes
60. No
61. Don’t know
62. Do you believe that malaria can cause disease?
63. Yes
64. No
65. Don’t know
66. Has anyone in your household been ill with fever or malaria in the last 12 month? If your answer is’ No or Don’t know skip to Q ’33’
67. Yes
68. No
69. Don’t know
70. How many members of your household were ill with fever or malaria in the last 12 months? Number ______________
71. Who was ill form the HH member? ________________________
72. What was the symptom you notice on? **Circle all that apply, do not prompt**
73. Difficult breath
74. Diarrhea
75. Headache
76. Chills/shivering
77. Loss of appetite
78. Rainy nose
79. Vomiting
80. Joint pain
81. Rash
82. Convulsion
83. Coma
84. Not symptom other than fever
85. Other ___________
86. Did you seek advice or treatment for the fever from any source? If your answer is ‘Yes’ skip to Q ‘28’ If Don’t know skip to Q ‘31’
87. Yes
88. No
89. Don’t know
90. What was the main reason for not taking for treatment? **Circle all that apply, do not prompt**
91. Health facilities are not available in the area
92. There are no HEWs in the kebelle
93. The cost for treatment too high
94. The available medicine are not effective to in curing malaria
95. There was no one to take Him/her for treatment
96. Don’t think it cures malaria
97. I don’t like it
98. Other____________________
99. Who provide the advice or treatment for the fever?
100. Household head
101. Self(ill member)
102. Other household member
103. HEWs
104. Health officer/nurse
105. Private clinic practitioner
106. Traditional healer
107. Faith healer
108. Other ______________________(specify)
109. Where was taken for treatments?
110. From governmental hospital
111. From Gov, health center
112. From Gov, health post
113. From NGO
114. From private clinic
115. Private pharmacy
116. Traditional pharmacy
117. others
118. How quick was take for treatment?
119. Within 24 Hrs
120. With 48Hrs
121. Within a weeks
122. After more than week
123. Don’t’ know
124. At any time during the illness, did take any drug for the illness? If your answer is ‘Yes’ skip to Q ‘33’
125. Yes (skip)
126. No
127. Don’t know (skip)
128. What was the main reason did not take drugs for the illness? **Circle all that apply, do not prompt**
129. Fever subsided
130. Drug was not prescribed
131. Prescribed drug was not available in the health post
132. Cost of drug was high
133. Referred but could not go to Health center
134. Other______________________________(specify)
135. What drug did take? **Circle all that apply do not prompt**
136. Choloroquine
137. Coartem
138. Quinine
139. Other malaria drug _____________
140. Traditional drug
141. Do you keep medicine at home for treating fever or malaria? if you ‘yes’ show me
142. Yes and seen
143. Yes and not seen
144. No
145. Don’t know
146. Have you ever heard of mosquito nets that can be used to cover your sleeping bed /place while sleeping? If your answer is ‘No’ skip to Q ‘47’
147. Yes
148. No
149. Do you think sleeping under the mosquito net protect from malaria disease?
150. Yes
151. No
152. Don’t know
153. Does your household have any mosquito nets that can be used while sleeping? If your answer is ‘No ‘skip to Q ‘46’ IF Don’t know skip to Q’47’
154. Yes
155. No
156. Don’t know
157. How many mosquito nets your households have?

Number __________________________

1. Can I see the mosquito net(s) in your house hold/
2. Observed
3. Not observed
4. Was any of the mosquito net actually hanging over a bed /mat?
5. Yes
6. No
7. Inspect the mosquito net for holes or tears ?
8. Good :no holes/tears
9. Damaged visible holes and tears
10. Where do you get the mosquito net ?
11. Gov, health facility
12. Private market
13. NGO
14. Other _________________
15. When do you get the mosquito net? Month________year E.C___________
16. Did anyone sleep under the mosquito net from the HH member last night? If your answer is ‘No” and “Don’t know” skip to Q “47”
17. Yes
18. No
19. Don’t know / no sure
20. Who slept under the mosquito nets last night? Skip to Q “47”
21. Children
22. Pregnant
23. women
24. all member
25. husband
26. What is the main reason for not having a mosquito net in your household?
27. Bed nets are not available in the area
28. The cost is too high
29. The available bed nets are not effective in preventing malaria
30. Don’t think it prevent malaria at all
31. We don’t like it
32. No malaria in the area
33. Other ____________________
34. Do you think that house spraying with chemical prevents malaria?
35. Yes
36. No
37. Don’t know
38. Was your house sprayed with chemicals to protect your family from getting malaria in the last 12 months?
39. Yes
40. No
41. Don’t know
42. Were all room sprayed?
43. All rooms
44. Some of the rooms
45. What was the importance of the spray and what does it protect?
46. From mosquito
47. From flies
48. From house bugs
49. Don’t know
50. Have you re-plastered your house in the last 12 month
51. yes
52. don’t know
53. When did the previous plastering take place? Month ago________________________
54. What is the main reason for not having a mosquito net in your household?
55. Bed nets are not available in the area
56. The cost is too high
57. The available bed nets are not effective in preventing malaria
58. Don’t think it prevent malaria at all
59. We don’t like it
60. No malaria in the area
61. Other ____________________
62. Do you think that house spraying with chemical prevents malaria?
63. Yes
64. No
65. Don’t know
66. Was your house sprayed with chemicals to protect your family from getting malaria in the last 12 months?
67. Yes
68. No
69. Don’t know
70. Were all room sprayed?
71. All rooms
72. Some of the rooms
73. What was the importance of the spray and what does it protect?
74. From mosquito
75. From flies
76. From house bugs
77. Don’t know
